# Incidence of herpes zoster in patients with altered immune function

**DOI:** 10.1007/s15010-013-0550-8

**Published:** 2013-11-10

**Authors:** S.-Y. Chen, J. A. Suaya, Q. Li, C. M. Galindo, D. Misurski, S. Burstin, M. J. Levin

**Affiliations:** 1 Evidera, Lexington, MA USA; 2 GlaxoSmithKline, Philadelphia, PA USA; 3 University of Colorado Denver, Anschutz Medical Campus, Aurora, CO USA

**Keywords:** Herpes zoster, Incidence, Immune-compromised, Persistent post-zoster pain

## Abstract

**Purpose:**

To estimate the incidence of herpes zoster (HZ) and rates of post-zoster pain in both the total study population and separately in patients with selected conditions/treatments associated with altered immune function.

**Methods:**

The health administrative claims databases for commercially insured, Medicare, and Medicaid populations, together accounting for approximately 51 million insured individuals, were analyzed between 2005 and 2009 in a retrospective cohort study. Incidence of HZ episodes per 1,000 person-years (PY) was estimated in all study populations as well as within nine potentially immune-altering conditions. Among patients with HZ, the 6-month rate of persistent post-zoster pain was estimated.

**Results:**

Analysis of 90.2 million PY at risk revealed that the incidence of HZ in the total study population was 4.82/1,000 PY. The incidence of HZ was highest among patients with bone marrow or stem cell transplant (43.03 %) followed by solid organ transplant, human immunodeficiency virus infection, and systemic lupus erythematosus [95 % confidence interval (CI) 15.19–17.41 %]. HZ incidence rates were higher among persons on immunosuppressants/chemotherapy than among non-users. In the total study population, HZ incidence increased with age (18–49 years: 3.37/1,000 PY; 65+ years: 8.43/1,000 PY; *P* < 0.01) and female gender (incidence ratio vs. male 1.39, 95 % CI 1.38–1.40 %). The 6-month rate of persistent post-zoster pain was 4.29 % (95 % CI 4.22–4.36 %), which was higher in patients with the selected conditions.

**Conclusions:**

Despite providing a relatively small fraction of overall HZ cases, persons with immune function-altering conditions make a large contribution to the societal healthcare burden because they have a higher risk of developing HZ and persistent post-zoster pain. These risk factors should be considered in HZ prevention efforts.

## Introduction

Herpes zoster (HZ) is caused by the reactivation in sensory ganglia of latent varicella-zoster virus (VZV), with clinical manifestations of an acute, painful dermatomal vesicular rash that can be followed by persistent post-zoster pain (postherpetic neuralgia) in the same dermatome [1]. HZ results in a significant economic burden in direct healthcare costs [2] and loss of productivity [3, 4]. The occurrence of post-zoster debilitating pain leads to higher direct healthcare costs [5] and adversely impacts the quality of life [6, 7].

Population studies in the USA in the early 1990s estimated the incidence of HZ to be 2.2/1,000 person-years (PY). [8]. In comparison, recent estimates of HZ incidence in the USA were 3–4/1,000 PY based on data up to 2005 [9–11]. Another USA study reported a trend toward increasing HZ incidence from 1.7/1,000 PY in 1993 to 4.4/1,000 PY in 2006 [12]. A similar observation was found in a recent study on the Medicare elderly population between 1991 (9.9/1,000 PY) and 2009 (13.8/1,000 PY) [13].

The onset of HZ is believed to result from declining VZV-specific cell-mediated immunity (CMI) [14], resulting in patients with conditions or treatments that alter immune function being more susceptible to HZ. The incidence of HZ has been examined in patients with various conditions or treatment scenarios that alter CMI, including solid organ transplant (SOT) [15–17], cancer [18, 19], human immunodeficiency virus (HIV) infection [20, 21], and autoimmune diseases, such as systemic lupus erythematosus (SLE) [18, 22, 23], rheumatoid arthritis (RA) [18, 24–26], and inflammatory bowel disease (IBD) [18, 27], or together as a group [9]. Although the risk of HZ has been evaluated in patients with individual conditions, a study that includes a large sample size from the general population enabling the assessment of the comparative risk of HZ among those conditions will be informative.

There is currently a licensed vaccine to help prevent HZ, as well oral medication that must be taken soon after its onset. Therefore, defining the risk of different populations could be helpful in implementing strategies to decrease the burden of HZ. The objective of this study was to estimate the incidence of HZ both in the total study population and separately in patients with selected conditions/treatments associated with altered CMI. Among patients with HZ, the rate of persistent post-zoster pain was also evaluated.

## Methods

### Data sources

The analysis was based on data extracted from three administrative medical and pharmacy claims databases on three large populations, namely, (1) commercially insured working adults and their dependents (approx. 30 million) from over 100 large self-insured employers and health plans; (2) Medicare-eligible retirees with Medicare supplemental insurance (approx. 3 million); (3) Medicaid beneficiaries from 12 states (approx. 22 million). The databases contained information on enrollment status, type of healthcare plan, and demographic characteristics, such as age, gender, and region of residence. The following information was captured on pharmacy claims: National Drug Code, dispensing date, quantity, days supplied, and plan- and patient-paid amounts. Details of healthcare service encounters were recorded on the medical claims, including date and place of service, provider type, plan- and patient-paid amounts, and the international classification of diseases, ninth revision, clinical modification (ICD-9-CM) diagnosis and procedure codes across all settings.

### Study populations

For inclusion in the study we first selected adults captured the databases aged ≥18 years between 01 January 2005 and 31 December 2009. We excluded individuals from the Medicare population aged <65 years and Medicaid enrollees aged ≥65 years, enrollees with dual eligibility of Medicaid and Medicare, and Medicaid enrollees lacking drug coverage because their medical and pharmacy claims were not completely captured in the database. To estimate the incidence of new episodes of HZ, we considered individuals with continuous medical and pharmacy benefits for a period of at least 6 months without any diagnosis of HZ (ICD-9-CM 053.xx). We also determined separately the incidence of HZ among patients with the following nine potentially CMI-altering conditions: cancer (excluding skin cancer), HIV infection, bone marrow or stem cell transplantation (BMSCT), SOT, SLE, RA, IBD, psoriasis, and multiple sclerosis (MS). These conditions were identified by ICD-9-CM diagnosis codes and procedures of the medical claims (Appendix Table 5). For cancer, we required at least two medical claims on two separate dates to ascertain a diagnosis. A patient could have multiple conditions and could also contribute to multiple disease populations. The follow-up for the total study population began after the subject had been diagnosed HZ free for 6 months (index date); the follow-up for the selected disease populations began after the subject had been diagnosed HZ free for at least 6 months and after the first disease-related claim.

### Ascertaining incident HZ cases

Herpes zoster episodes were ascertained based on principal or secondary diagnosis codes of 053.0–053.11, 053.14, 053.19, 053.2x–053.9x. we excluded two post-herpetic neuralgia-related diagnoses (053.12, 053.13) to prevent mis-classification of prevalent HZ cases.

### Identification of persistent post-zoster pain

Patients with HZ who had at least 6 months continuous enrollment following the incident HZ diagnosis were assessed for persistent post-zoster pain, which was defined in this study as: (1) a diagnosis of post-herpetic trigeminal neuralgia (ICD-9-CM 053.12) or post-herpetic polyneuropathy (ICD-9-CM 053.13), (2) a diagnosis of HZ with other nervous system complications (ICD-9-CM 053.19) and more than 30-day supply of medications commonly used for post-zoster pain (Appendix Table 6 ), or (3) unspecified neuralgia, neuritis, or radiculitis (ICD-9-CM 729.2) if it first appeared after the HZ diagnosis.

### Patient characteristics

Age and gender were assessed at the index date. Chemotherapy treatments among patients with cancer and the administration of immunosuppressants for the selected conditions, except for HIV-infected individuals, were recorded from pharmacy claims and procedure codes in medical claims during the study period (Appendix Table 7).

### Statistical analysis

To calculate the incidence of HZ, enrollees were followed from the index date to the end of their continuous enrollment or the date of the incident HZ episode, whichever came first. The incidence of HZ was estimated as the number of incident HZ cases divided by the person-time at risk and presented as number of cases/1,000 PY. The 95 % confidence interval (CI) of incidence was estimated assuming a Poisson distribution.

Incidence of HZ was reported by age (age categories: 18–49, 50–59, 60–64, 65+ years) and by gender. Since not all selected conditions are immunocompromising when untreated, we also estimated the incidence of HZ by use of immunosuppressants/chemotherapies. The incident rate ratio (IRR) of HZ between these mutually exclusive subgroups was reported, and the 95 % CI of IRR was estimated based on the Mantel–Haenszel combined estimate. The Mantel extension of the Armitage–Cochran trend test was used to assess the linear trend in incidence rate from age group 18–49 to 65+ years. Among patients with HZ who had at least 6 months of follow-up, we estimated the 6-month rates of persistent post-zoster pain. Analyses were conducted using SAS ver. 9.2 (SAS Institute, Cary, NC).

## Results

### Characteristics of study sample

There were 218,025,906 enrollees available in the Commercial, Medicare and Medicaid MarketScan database from 2005 to 2009. Among these, 51,022,838 enrollees met the inclusion/exclusion criteria. The mean age of the total study population was 43 years, 54.0 % were female, and the average follow-up was 1.8 years (Table 1). The mean age of the selected conditions was the lowest for patients with HIV infection (mean age 42 years) and the highest for patients with cancer (mean age 60 years). Use of immunosuppressants was most common among patients with SOT (67.4 %) or BMSCT (58.6 %), followed by RA (49.4 %), SLE (47.0 %), IBD (37.8 %), MS (33.0 %), and psoriasis (30.3 %). Of the cancer patients, 28.7 % had pharmacy claims for chemotherapies.

**Table 1:** Characteristics of the study sample

Characteristics of the study sample	Number of persons	Age (years)		Age group (%)				Sex		Use of immunosuppressants/chemotherapy (%)
		Mean	SD	18–49 years	50–59 years	60–64 years	65+ years	Male	Female	
Total study population	51,022,838	43.1	15.8	66.0	19.2	6.2	8.6	46.0	54.0	N/A
Bone marrow or stem cell transplant	14,679	49.3	12.8	42.8	35.3	15.5	6.5	52.4	47.6	58.6
Solid organ transplant	61,189	49.7	12.5	44.5	34.0	13.1	8.4	59.2	40.8	67.4
Human immunodeficiency virus infection	121,956	41.8	10.6	76.8	18.8	3.2	1.2	69.8	30.2	N/A
Systemic lupus erythematosus	144,137	46.9	13.2	56.7	27.5	8.7	7.1	12.2	87.8	47.0
Rheumatoid arthritis	571,555	52.7	13.9	39.4	31.8	12.7	16.1	26.7	73.3	49.4
Cancer	1,462,356	59.5	13.8	21.8	30.1	16.6	31.5	45.1	54.9	28.7
Inflammatory bowel disease	345,565	47.0	15.0	55.5	25.1	9.2	10.2	43.5	56.5	37.8
Multiple sclerosis	146,261	46.3	12.1	60.1	27.5	7.2	5.2	24.1	75.9	33.0
Psoriasis	536,770	46.2	14.7	57.2	24.8	9.1	8.9	45.6	54.4	30.3

SD, Standard deviation; N/A, not available

### Incidence rate of HZ

In the total study population, 435,378 new cases of HZ occurred during 90,236,779 PY (Table 2), indicating an incidence of 4.82/1,000 PY (95 % CI 4.81–4.84). The incidence of HZ among the selected conditions was higher than that in the total study population (Table 2). The incidence of HZ was highest among patients with BMSCT (43.03/1,000 PY; 95 % CI 39.96–46.28; ninefold higher than that of the total study population). Patients with SOT (17.04/1,000 PY; 95 % CI 16.23–17.88), HIV (17.41/1,000 PY; 95 % CI 16.81–18.01) and SLE (15.19/1,000 PY; 95 % CI 14.69–15.69) had HZ incidence rates over threefold higher than that in the total study population. The incidence of HZ in the remaining evaluated conditions was between two- and threefold higher than that of the total study population, including RA (12.24/1,000 PY; 95 % CI 12.02–12.47), cancer (11.70/1,000 PY; 95 % CI 11.57–11.83), IBD (9.31/1,000 PY; 95 % CI 9.06–9.56), MS (8.60/1,000 PY; 95 % CI 8.24–8.96) and psoriasis (8.03/1,000 PY; 95 % CI 7.84–8.22).

**Table 2:** Incidence of herpes zoster by various immune-altering conditions

Characteristics of the study sample	Number of herpes zoster cases	Person-years	Incidence^a^	95 % Confidence interval	Incidence by use of immunosuppressants or chemotherapy			
					Users		Non-users	
					Incidence^a^	95 % CI	Incidence^a^	95 % CI
Total study population	435,378	90,236,779	4.82	4.81–4.84	N/A	**–**	N/A	**–**
Bone marrow or stem cell transplant	726	16,870	43.03	39.96–46.28	51.50	(47.18–56.11)	30.23	26.21–34.68
Solid organ transplant	1,673	98,173	17.04	16.23–17.88	18.85	(17.86–19.89)	12.11	10.82–13.52
Human immunodeficiency virus infection	3,207	184,198	17.41	16.81–18.01	N/A	–	N/A	–
Systemic lupus erythematosus	3,540	233,096	15.19	14.69–15.69	17.85	(17.11–18.61)	12.23	11.58–12.90
Rheumatoid arthritis	11,446	934,811	12.24	12.02–12.47	14.28	(13.96–14.61)	9.64	9.34–9.94
Cancer	29,698	2,538,706	11.70	11.57–11.83	15.63	(15.33–15.93)	10.25	10.11–10.40
Inflammatory bowel disease	5,257	564,563	9.31	9.06–9.56	12.07	(11.64–12.51)	7.15	6.86–7.45
Multiple sclerosis	2,146	249,551	8.60	8.24–8.96	11.00	(10.35–11.68)	7.05	6.64–7.49
Psoriasis	6,927	862,544	8.03	7.84–8.22	10.85	(10.49–11.23)	6.47	6.26–6.68

HZ, Herpes zoster; CI, confidence interval

^a^Incidence presented as number of cases per 1,000 person-years (PY)

### Incidence rate ratio of HZ for patients receiving immunosuppressants or chemotherapy

**Fig. 1:**
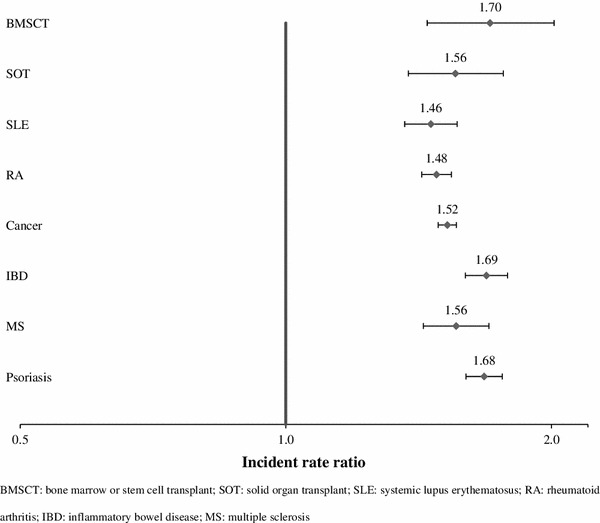
Incidence rate ratio of herpes zoster (HZ) for users of immunosuppressants or chemotherapy (users vs. non-users). *BMSCT* Bone marrow or stem cell transplant, *SOT* solid organ transplant, *SLE* systemic lupus erythematosus, *RA* rheumatoid arthritis, *IBD* inflammatory bowel disease, *MS* multiple sclerosis

Across the selected conditions, the incidence rates of HZ were higher among users of immunosuppressants or chemotherapy than among non-users (Table 2). Figure 1 illustrates the IRR with 95 % CI of users versus non-users. Irrespective of the underlying conditions, users of immunosuppressant or chemotherapy had an approximately 50 % higher risk of HZ than those without these interventions. The increased risk of HZ associated with immunosuppressants or chemotherapy was highest among patients with BMSCT (IRR 1.70, 95 % CI 1.45–2.01) and lowest among patients with SLE (IRR 1.46, 95 % CI 1.36–1.56).

### Incidence rate and rate ratio of HZ by age and gender

As shown in Table 3, the incidence rate of HZ in the total study population increased significantly with age (*P* < 0.01) from 3.37/1,000 PY (95 % CI 3.35–3.38) for those aged 18–49 years to 8.43/1,000 PY for those aged 65+ years (95 % CI 8.37–8.49). Compared to individuals aged 18–49 years, the HZ incidence rate was 91 % higher (IRR 1.91, 95 % CI 1.90–1.92) among those aged 50–59 years, 129 % higher (IRR 2.29, 95 % CI 2.27–2.31) among those aged 60–64 years and 150 % higher among those aged 65+ years (IRR 2.50, 95 % CI 2.48–2.52). An increasing HZ incidence with age was also observed among patients with SOT, SLE, RA, cancer, IBD, MS, and psoriasis (all *P* < 0.01). However, except for persons with psoriasis, the risk of HZ in these conditions was less influenced by age (as suggested by the IRRs toward the null) than in the total study population. Among patients with BMSCT, there was no significant increase in HZ incidence with age (Table 3). A decreasing trend of incidence rate was observed among patients with HIV (*P* < 0.01), from 17.8 to 16.0/1,000 PY among those aged <65 years to 10.70/1,000 PY (95 % CI 6.62–16.35) in those aged 65+ years.

**Table 3:** Incidence and rate ratio of herpes zoster by age group

Characteristics of the study population	Incidence^a, b^ (95 % CI)								Incidence rate ratio (95 % CI)					
	18–49 years		50–59 years		60–64 years		65+ years		50–59 vs. 18–49		60–64 vs. 18–49		65+ vs. 18–49	
Total study population	3.37 (3.35–3.38)		6.43 (6.40–6.47)		7.71 (7.64–7.79)		8.43 (8.37–8.49)		1.91 (1.90–1.92)		2.29 (2.27–2.31)		2.50 (2.48–2.52)	
Bone marrow or stem cell transplant	40.20 (35.6–45.12)		43.22 (38.2–48.65)		50.71 (41.8–60.92)		44.73 (33.2–58.97)		1.08 (0.91–1.27)		1.26 (1.01–1.57)		1.11 (0.81–1.51)	
Solid organ transplant	13.30 (12.2–14.43)		19.41 (17.9–20.91)		19.76 (17.2–22.56)		23.15 (19.9–26.76)		1.46 (1.30–1.63)		1.49 (1.27–1.74)		1.74 (1.46–2.06)	
Human immunodeficiency virus infection	17.83 (17.1–18.55)		16.42 (15.1–17.75)		16.02 (12.8–19.78)		10.70 (6.62–16.35)		0.92 (0.84–1.01)		0.90 (0.72–1.11)		0.60 (0.37–0.92)	
Systemic lupus erythematosus	13.39 (12.7–14.04)		15.40 (14.5–16.35)		20.01 (18.0–22.18)		23.39 (21.0–25.91)		1.15 (1.07–1.24)		1.49 (1.33–1.67)		1.75 (1.56–1.96)	
Rheumatoid arthritis	8.32 (8.02–8.63)		12.75 (12.3–13.15)		15.31 (14.5–16.08)		18.34 (17.6–19.04)		1.53 (1.46–1.61)		1.84 (1.73–1.96)		2.20 (2.09–2.32)	
Cancer	8.39 (8.15–8.64)		10.94 (10.7–11.17)		13.05 (12.6–13.42)		14.14 (13.8–14.40)		1.30 (1.26–1.35)		1.55 (1.49–1.62)		1.68 (1.63–1.74)	
Inflammatory bowel disease	6.89 (6.59–7.19)		11.02 (10.5–11.55)		11.67 (10.7–12.69)		15.48 (14.4–16.53)		1.60 (1.50–1.71)		1.69 (1.54–1.86)		2.25 (2.08–2.43)	
Multiple sclerosis	6.83 (6.42–7.27)		10.75 (10.0–11.52)		11.91 (10.2–13.75)		12.35 (10.4–14.50)		1.57 (1.43–1.73)		1.74 (1.48–2.04)		1.81 (1.51–2.15)	
Psoriasis	5.28 (5.08–5.49)		9.46 (9.08–9.86)		13.11 (12.2–13.99)		15.39 (14.5–16.27)		1.79 (1.69–1.90)		2.48 (2.30–2.68)		2.91 (2.72–3.12)	

^a^Incidence rate presented as number of cases per 1,000 PY

^b^Linear trend of incidence rate was significant at *P* < 0.01 except for bone marrow or stem cell transplant (*P* = 0.08)

**Fig. 2:**
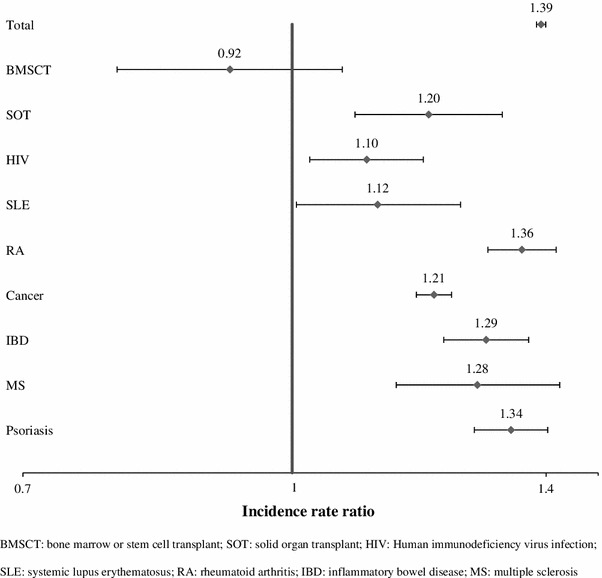
Incidence rate ratio of HZ for gender (female vs. male). *HIV* Human immunodeficiency virus infection

In the total study population (Fig. 2), the incidence rate of HZ among females was 39 % higher than that of males (IRR 1.39, 95 % CI 1.38–1.40). Among patients with BMSCT, the HZ incidence rate was not significantly different between females and males (IRR 0.92, 95 % CI 0.79–1.07), but the incidence rate of HZ among females was higher than males in the other conditions, with IRRs ranging from 1.10 (95 % CI 1.02–1.19) in HIV to 1.36 (95 % CI 1.30–1.42) in RA.

### Six-month rate of persistent post-zoster pain

There were 322,877 patients who had >6 months of continuous enrollment after the onset of HZ to provide an estimate of the 6-month rates of persistent post-zoster pain (Table 4). Overall, the 6-month rate was 4.29 % (95 % CI 4.22–4.36). In general, the rate was higher among patients with the selected conditions than among the total study population, with BMSCT being the highest (10.18 %, 95 % CI 7.83–13.14). For the other selected immunocompromised conditions, the rate of persistent post-zoster pain ranged from 5.08 % among patients with psoriasis (95 % CI 4.51–5.73) to 7.22 % among patients with RA (95 % CI 6.68–7.80).

**Table 4:** Six-month rate of persistent post-zoster pain among patients with herpes zoster

Characteristics of the study population	Herpes zoster cases (*n*)	Rate of persistent post-zoster related pain (%)	95 % CI
Total study population	322,877	4.29	4.22–4.36
Bone marrow or stem cell transplant	501	10.18	7.83–13.14
Solid organ transplant	1,205	6.72	5.44–8.28
Human immunodeficiency virus infection	2,311	6.10	5.20–7.15
Systemic lupus erythematosus	2,593	6.44	5.56–7.45
Rheumatoid arthritis	8,294	7.22	6.68–7.80
Cancer	21,620	7.03	6.70–7.38
Inflammatory bowel disease	3,793	5.85	5.15–6.65
Multiple sclerosis	1,563	5.57	4.53–6.82
Psoriasis	4,978	5.08	4.51–5.73

## Discussion

The objectives of this study were to estimate the incidence of HZ and the rate of persistent post-zoster pain within 6 months after the incident HZ event among adults in the total study population and in those with selected potentially CMI-altering conditions. The relative impact of age, gender and exposure to immunosuppressants or chemotherapies on the incidence of HZ were also assessed. Using a large nation-wide, multiple-payer U.S. administrative claims database, we found a HZ incidence of 4.82/1,000 PY for the total study population. A higher incidence was observed associated with female gender and older age. The incidence of HZ was highest among patients with BMSCT, followed by SOT, HIV, and SLE (43.03–15.19/1,000 PY). The other conditions (RA, cancer, IBD, MS, and psoriasis) had an incident two- to threefold that of the total study population (12.24–8.03/1,000 PY).

As both the incidence of HZ and the prevalence of risk factors change over time, it is important to assess the most current disease burden. Therefore, this study, derived from 51 million people participating in three different healthcare insurance programs between 2005 and 2009, provides recent data on the epidemiology of HZ in the USA. Although our study population might not be directly comparable to those of previous studies due to differences in data sources and patient profiles (e.g., age and gender), the higher incidence of HZ (4.82/1,000 PY) observed in our study is consistent with an increasing trend of HZ incidence over time [8–13].

Within the nine selected conditions, the risk of HZ was two- to tenfold greater than that seen in the total study population. Although it is difficult to compare absolute values from past studies given the differences in methodology, populations, and time period, our findings are consistent those of earlier studies in that there is a strong effect of immune suppression on the risk of HZ [9, 18, 19, 23, 25, 27]. In our study, a more accurate comparison across conditions was possible because patients with these selected conditions were drawn from one large population using the same methodology. Although the majority of HZ cases occurred among individuals without concurrent alteration in immune function [9, 11], the higher risk of HZ among patients with altered immune function may be more burdensome from an economic perspective. For example, the higher rate of persistent post-zoster pain observed in these patients will likely require significant healthcare resources and would decrease their productivity and/or quality of life. The comparative risk estimates observed will be informative for the clinicians and payers in targeting populations at a higher risk.

This study also evaluated the comparative effect of various factors across these high-risk conditions. Consistent with the literature [8–12, 28], our study found a higher risk of HZ associated with older age in the total study population. Among the selected conditions there was an increasing age trend for HZ except for BMSCT and HIV infection. However, it is worth noting that the absolute risk ratio of HZ associated with older age in subjects with these conditions is much smaller relative to that in the total study population. This could be because the increased risk associated with older age (mediated by declining VZV-specific CMI) is relatively small compared with that associated with strong immune suppressive effects (on VZV-specific CMI) of the selected diseases and their therapies. The unexpected decreasing risk of HZ associated with older age in HIV-infected people in our study appears to be driven by the lower risk among patients aged ≥65 years (IRR = 0.60 compared to patients aged 18–49 years; 95 % CI 0.37–0.92). A possible explanation is that older patients who do survive with HIV might be relatively more immune competent and more likely to adhere to treatment than younger patients.

As has been observed in many earlier studies [9, 11, 12, 29–37]—but not all [8, 18, 24, 38, 39]—we found a higher risk of HZ associated with female gender. No biological explanation has been proposed for this finding. Since we used an administration health claims data, the behavioral differential in health-seeking behavior could not be ruled out. Interestingly, no gender effect in patients with BMSCT was found among our study population, which supports our hypothesis regarding the declining role of other risk factors when a strong immune suppressive effect is present. Our study also confirmed the higher risk associated with immunosuppressants and chemotherapies previously reported [19, 22, 24, 26, 37].

Post-herpetic neuralgia is the most common complication of HZ. The authors of two studies reported that 18–27 % of the adult patients with HZ in their studies had persistent pain beyond 90 days [11, 40]. This is substantially lower (4.29 % for persistent post-zoster pain) than that in our study. However, the rate of post-zoster pain in this article is not equivalent to that of the more usually reported post-herpetic neuralgia, as we used an algorithm combining diagnostic codes and the utilization of pain-related medications to identify patients likely to have experienced persistent post-zoster pain. Thus, it remains challenging to accurately identify patients experiencing persistent post-zoster pain using the ICD-9-CM diagnosis system, which does not have a specific post-herpetic neuralgia/diagnosis code. Assuming that the disease-specific effects on this algorithm are random (i.e., had the same ability to discriminate this condition between the sub-groups even if there was any mis-classification), the relative rates between populations is informative. Our findings of a higher rate of persistent post-zoster pain in the evaluated conditions relative to the total study population indicate that persistent post-zoster pain occurs even when the inflammatory response is blunted.

This study has several structural limitations. Diagnoses were derived from administrative billing records which are subject to mis-coding or under-coding and which are not validated against medical charts. Moreover, we only captured health encounters that were reimbursed by the plan. Hence, our data source did not capture the individuals developing HZ/persistent post-zoster pain who did not seek healthcare. Healthcare services or prescription medications paid completely out-of-pocket or by other supplemental insurance were not observed, as were any medicines that had been administered on experimental protocols. Exposure to immunosuppressants, chemotherapies, and pain-related medications could be mis-classified if they were not reimbursed by the health plan or patients did not actually receive the treatment. For example, the use of immunosuppressants appeared to be lower than anticipated in SOT patients; such mis-classification could lead to biased estimates in either direction. The estimated incidence within the conditions with altered immune function were also subject to detection bias, since these patients had more frequent healthcare encounters, which would bias the comparative results between the total study population and the immune-suppressed patients. Finally, our findings might not be generalizable to other populations such as un-insured or veterans.

## Conclusions

In this study we combined three large databases to assess the current epidemiology of HZ. Despite providing a fraction of overall HZ cases, persons with immune function-altering conditions have a higher risk of developing HZ and persistent post-zoster pain. Older age and female gender were also associated with higher risks. These risk factors should be considered in the design of preventive programs that include the use of safe and effective HZ vaccines.

## Appendix

Tables 5, 6 and 7.

**Table 5:** International classification of diseases, ninth revision, clinical modification codes of conditions in the study

Disease	ICD-9-CM diagnosis codes/procedure codes	Description
Cancer	140.xx–171.xx, 174.xx–208.xx	Malignant neoplasm except for other malignant neoplasm of skin (172.xx, 173.xx)
	99.25, 99.28	Injection or infusion of cancer chemotherapeutic substance or biological response modifier as an antineoplastic agent
Human immunodeficiency virus infection	042, 079.53, V08	Human immunodeficiency virus infection
Solid organ transplant	V42.0, 55.6x	Kidney transplant
	V42.1, 33.6, 37.51	Heart transplant
	V42.6, 33.5x, 33.6	Lung transplant
	V42.7, 50.5x	Liver transplant
Bone marrow or stem cell transplant	V42.81, V42.82, 41.0x	Bone marrow or stem cell transplant
Rheumatoid Arthritis	714.xx	Rheumatoid arthritis
Inflammatory bowel disease	555.x	Regional enteritis
	556.x	Ulcerative colitis
Systemic lupus erythematosus	710.0	Systemic lupus erythematosus
Multiple sclerosis	340.x	Multiple sclerosis
Psoriasis	696.x	Psoriasis and similar disorders

ICD-9-CM, International classification of diseases, ninth revision, clinical modification

**Table 6:** Medications for persistent post-zoster-related pain

Opioids	Codeine, hydrocodone, oxycodone, pentazocine, propoxyphene, tramadol, fentanyl, hydromorphone, meperidine, morphine, butorphanol, oxymorphone, levorphanol, methadone
Other analgesic medications	Acetaminophen and combinations, aspirin, and combinations
Nonsteroidal anti-inflammatory drugs	Ibuprofen and combinations, naproxen and combinations, indomethacin, diclofenac, nabumetone, etodolac, meloxicam, sulindac, piroxicam, oxaprozin, ketoprofen, tolmetin, ketorolac tromethamine, celecoxib, valdecoxib, diflunisal
Anticonvulsants	Gabapentin, pregabalin
Lidoderm	Lidocaine patch 5 %
Other topical anesthetic	Capsaicin-topical
Serotonin-norepinephrine reuptake inhibitors (SNRI)	Duloxetine
Tricyclic antidepressants (TCA)	Amitriptyline and combinations, desipramine, nortriptyline doxepin, imipramine

**Table 7:** Immunosuppressants and chemotherapies recorded from pharmacy claims and procedure codes during the study period

Immunosuppressants	Cyclosporine, tacrolimus, everolimus, sirolimus, azathioprine, mycophenolic acide, mycophenolate mofetil, dexamethasone (oral), hydrocortisone (oral), prednisolone (oral), prednisone (oral), methylprednisolone (oral), triamcinolone (oral), anti-lymphocyte and anti-thymocyte globulin, basiliximab, canakinumab, certolizumab pegol, daclizumab, golimumab, infliximab, muromonab, natalizumab, omalizumab, rituximab, tocilizuma, ustekinumab, adalimumab, etanercept, abatacept, alefacept, rilonacept
Chemotherapies	Aldesleukin, alemtuzumab, altretamine, arsenic trioxide, asparaginase, azacitidine, bendamustine, bevacizumab, bexarotene, bleomycin, bortezomib, busulfan, cabazitaxel, capecitabine, carboplatin, carmustine, cetuximab, chlorambucil, cisplatin, cladribine, clofarabine, cyclophosphamide, cytarabine, dactinomycin, dasatinib, daunorubicin, decitabine, denileukin diftitox, docetaxel, doxorubicin, dromostanolone, eculizumab, epirubicin, erlotinib, estramustine, etoposide, everolimus, floxuridine, fludarabine, fluorouracil, gemcitabine, gemtuzumab, ozogamicin, hydroxyurea, ibritumomab, idarubicin, ifosfamide, ifosfamide/mesna, imatinib, irinotecan, ixabepilone, lomustine, masoprocol, mechlorethamine, megestrol, melphalan, mercaptopurine, methotrexate, methoxsalen, mitomycin, mitotane, mitoxantrone, nelarabine, nilotinib, ofatumumab, oxaliplatin, paclitaxel, panitumumab, pazopanib, pegaspargase, pemetrexed, pentostatin, pipobroman, plicamycin, porfimer, pralatrexate, procarbazine, ranibizumab, rituximab, romidepsin, sorafenib, streptozocin, sunitinib, temozolomide, temsirolimus, teniposide, thioguanine, thiotepa, topotecan, trastuzumab, tretinoin, valrubicin, vinblastine, vincristine, vinorelbine, vorinostat
